# Analysis of the high-dose-range radioresistance of prostate cancer cells, including cancer stem cells, based on a stochastic model

**DOI:** 10.1093/jrr/rrz011

**Published:** 2019-04-29

**Authors:** Ryo Saga, Yusuke Matsuya, Rei Takahashi, Kazuki Hasegawa, Hiroyuki Date, Yoichiro Hosokawa

**Affiliations:** 1 Department of Radiation Sciences, Division of Medical Life Sciences, Hirosaki University Graduate School of Health Sciences, 66-1 Hon-cho, Hirosaki Aomori, Japan; 2 Graduate School of Health Sciences, Hokkaido University, Kita-12 Nishi-8, Kita-ku, Sapporo, Hokkaido, Japan; 3 Nuclear Science and Engineering Center, Research Group for Radiation Transport Analysis, Japan Atomic Energy Agency (JAEA), 2-4 Shirakata, Tokai, Ibaraki, Japan; 4 Faculty of Health Science, Hokkaido University, Kita-12 Nishi-8, Kita-ku, Sapporo, Hokkaido, Japan

**Keywords:** cell-killing model, radioresistance, cancer stem cell, dose–response curve, BED

## Abstract

In radiotherapy, cancer stem cells (CSCs) are well recognized as one of the radioresistant cell types. Even in a small subpopulation, CSCs may have an influence on tumor control probability, represented by cell killing after irradiation. However, the relationship between the percentage content of CSCs and the cell survival dose–response curve has not yet been quantitatively clarified. In this study, we developed a cell-killing model for two cell populations (CSCs and progeny cells) to predict the surviving fractions, and compared it with the conventional linear–quadratic (LQ) model. Three prostate cancer cell lines (DU145, PC3 and LNCaP) were exposed to X-rays at doses ranging from 0 to 10 Gy. After the irradiation, we performed clonogenic survival assays to generate the cell survival curves, and carried out flow-cytometric analyses to estimate the percentage content of CSCs for each cell line. The cell survival curves for DU145 cells and PC3 cells seemed not to follow the conventional LQ model in the high dose range (>8 Gy). However, the outputs of the developed model agreed better with the experimental cell survival curves than those of the LQ model. The percentage content of CSCs predicted by the developed model was almost coincident with the measured percentage content for both DU145 cells and PC3 cells. The experiments and model analyses indicate that a small subpopulation of radioresistant CSCs has lower radiosensitivity in the high-dose range, which may lessen the clinical outcome for patients with prostate cancer after high-dose radiation therapy.

## INTRODUCTION

In the application of radiotherapy, such as intensity-modulated radiation therapy, cancer cells with radioresistance can allow relapse or distant metastasis, and a poor prognosis [1, 2]. A small subpopulation of the cells called cancer stem cells (CSCs) has been investigated previously, and they exist in almost all carcinomas. In recent decades, CSCs have attracted attention as a therapeutic target, because of their metastatic potential and radioresistance [3, 4]. The stem cells in a tumor can be experimentally detected by using the expression of surface antigens such as cluster of differentiation (CD) 44, CD133 and aldehyde dehydrogenase 1 (a cytosolic detoxifying enzyme), known as the CSC markers for various types of cancer cells [4–9]. Increased expression of the CSC markers is closely related to poor prognosis [10], and has been clinically used for predicting the degree of radioresistance in radiotherapy [11].

The linear–quadratic (LQ) model, which quantitatively describes the relationship between absorbed dose and fraction of cells surviving, is widely used for determining the treatment planning in multifractionated radiotherapy [12]. Although the LQ model is preferred for reasons of simplicity and consistency with cell experiments and clinical outcomes, the model does not explicitly include radiosensitive factors, such as dose-rate effects and cell-cycle dependency [13–15]. Several cell-killing models considering radiosensitive factors (such as microdosimetry, dose-rate effects and intercellular communication) have been proposed for providing the relationship between absorbed dose and cell survival [16–22]. Among these, a trial for modeling cell-killing in multicell populations was also performed [20, 21], indicating that some modifications of the dose–response curve were required. However, the relationship between measured CSC contents and the cell survival curve described by the model has not been quantified yet. Furthermore, it seems to be difficult to measure clonogenic survival of pure CSCs in terms of *in vitro* experiments. So, a biologically mechanistic cell-killing model adaptable for radiotherapy is necessary for providing an analysis tool for CSCs in radiation biology and for precision of tumour control probability in radiation therapy.

In our previous *in vitro* experiments, the clonogenicity of the three types of prostate cancer (PCa) cell lines (i.e. PC3, DU145 and LNCaP) after exposure to the high dose of 10 Gy exhibited lower radiosensitivity than predicted for low-dose cell survival by using the LQ model (Murata *et al.*, unpublished data). Due to the unexpected radioresistance in the high dose range, the accuracy of fitting the model predictions to experimental data is sometimes poor. Regarding recent reports on the CSCs [3, 4], we can hypothesize that the subpopulations of the CSCs showing radioresistance might be involved in the discrepancy between the measured value and the cell survival estimated by the LQ model. So, our interest was focused on measuring the CSC fraction and quantifying the involvement of CSCs in the cell survival curve by using a mathematical model.

In this study, to mechanistically investigate the impact of the CSCs on the cell survival (dose–response) curve, we performed a multilateral analysis using *in vitro* experiments and the stochastic model taking the CSC fraction into account. Finally, we showed the lower radiosensitivity of the progeny cells (PCs) in the high dose range to be attributable to a small percentage of the CSCs.

## MATERIALS AND METHODS

### Biological experiments for the cell survival curve and the CSC fraction

#### Reagents

Phycoerythrin (PE)-conjugated monoclonal mouse anti-human CD133 (Catalog no. 372803) and mouse IgG1, κ isotype control (Catalog no. 400114), as well as fluorescein isothiocyanate (FITC)-conjugated monoclonal mouse anti-human CD44 (Catalog no. 338803), and mouse IgG1, κ isotype control (Catalog no. 400107) were purchased from BioLegend, Inc. (Tokyo, Japan).

#### Cell culture

The human PCa cell lines PC3 (bone metastatic cell line), DU145 (brain metastatic cell line) and LNCaP (lymph node metastatic cell line) were purchased from RIKEN Science Institute BRC (Ibaraki, Japan). The cells were maintained at 37°C in a 5% CO_2_ environment in RPMI 1640 medium (Thermo Fisher Scientific Inc. Tokyo, Japan) supplemented with 10% heat-inactivated fetal bovine serum (FBS) (Japan Bioserum Co. Ltd, Hiroshima, Japan) and 1% penicillin/streptomycin (Wako Pure Chemical Industries, Ltd, Osaka, Japan).

#### Irradiation conditions

The cultured cells were irradiated with kilo-voltage X-rays (150 kVp, 1.0 Gy/min) through a 0.5 mm aluminum and 0.3 mm copper filter using an X-ray generator (MBR-1520R-3; Hitachi Medical Co. Ltd, Tokyo, Japan), at a distance of 45 cm from the target. The dose-averaged linear energy transfer (LET) was estimated to be 1.53 keV/μm, which was calculated by Particle and Heavy Ion Transport code System (PHITS) ver. 3.02 [24]. The dose in air was monitored with a thimble ionization chamber placed next to the sample during irradiation.

#### Clonogenic survival assay

The clonogenic potency was obtained by means of a colony formation assay. The appropriate number of cells were seeded on the φ60 culture dish immediately after the X-ray irradiation. The cells were fixed with methanol (Wako Pure Chemical Industries, Ltd) 10–20 days after irradiation, and stained with Giemsa staining solution (Wako Pure Chemical Industries, Ltd). Colonies including >50 cells were counted. The surviving fraction for each cell line was calculated from the ratio of the plating efficiency for irradiated cells to that for non-irradiated cells.

#### Flow cytometric analysis for detecting the CSCs

To analyze the expression of the CSC markers, the cells were incubated in 100 μl phosphate-buffered saline without calcium chloride or magnesium chloride (PBS (–), TAKARA BIO INC.) containing 5% FBS and FITC anti-human CD44 (3 μl/10^6^ cells) and PE anti-human CD133 (3 μl/10^6^ cells) or respective mouse IgG1 isotype control antibodies (3 μl/10^6^ cells) for 15 min at 4°C in the dark. After staining, the cells were centrifuged, resuspended in PBS (–), and analyzed by direct immunofluorescence flow cytometry using a BD FACS Aria™ Cell Sorter (BD Biosciences, Ltd, Tokyo, Japan).

### Application of cell-killing model for describing the cell survival curve

#### Linear–quadratic model

To compare the cell survival curve with the proposed model, the LQ model was first applied to the experimental cell survival. The formula of the LQ model is expressed by

(1) $$\;-\;\ln\;S=\alpha D+\beta D^{2},$$

where *S* is the surviving fraction of cells, and *α* and *β* are the proportionality factors of the absorbed dose (*D*) (Gy^−1^) and the dose squared (*D*^*2*^) (Gy^−2^), respectively.

#### Integrated cell-killing model considering the CSC fraction

To investigate the contribution of CSCs to the dose–response curve, we developed a cell-killing model composed of two cell populations based on the integrated microdosimetric-kinetic (IMK) model for acute irradiation [13, 15, 22]. The IMK model enables us to evaluate the impact of intercellular communication as well as microdosimetry on the dose–response curve, so we selected the IMK model not the LQ model for developing the integrated modeling. Using the part of the DNA-targeted effects (DNA-TEs) in the IMK model [22] (the same formula as the MK model [17]), the surviving cell fraction can be given by

(2) $$-\;\ln\;S=\left(\alpha_{0}+\;\frac{y_{D}}{\rho \pi r_{d}^{2}}\beta_{0}\right)D+\;\beta_{0}D^{2},$$

where *α*_0_ and *β*_0_ are the proportionality factors of the *D* in Gy^−1^ and the *D*^*2*^ in Gy^−2^, respectively, *y*_*D*_ is the dose-mean lineal energy in keV/μm, which represents the track structure of ionizing radiation in terms of microdosimetry [23–25], *ρ* and *r*_d_ are the density of water (1.0 g/cm^3^) and the diameter of the target packaged in the cell nucleus (set to be 1.0 μm in this study). The derivation process of the IMK model for the DNA-TEs was summarized in previous reports [22]. Equation 2 considers the microdosimetry *y*_*D*_ in the cell survival formula.

**Fig. 1.**
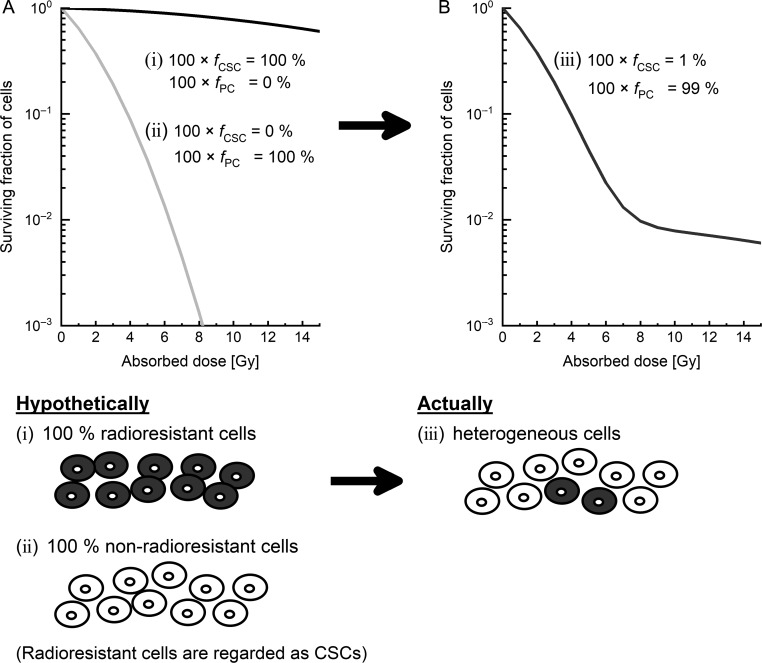
Schematic of the proposed model in this study. (A) The dose–response curve hypothetically dividing two types of cell populations from one type of cell line: (i) a group including only radioresistant cells, and (ii) another group consisting of only non-radioresistant cells. (B) The dose–response curve of actual heterogeneous cell lines (iii), which include 1% radioresistant cells. In this study, radioresistant cells were regarded as CSCs. CSCs: cancer stem cells.

Next, we considered the cultured cell populations, including the PCs fraction of *f*_PC_ and the CSCs fraction of *f*_CSC_ (= 1 − *f*_PC_) during irradiation. The schematic representation is shown in Fig. 1, in which the radiosensitivity of the CSCs is lower than that of the PCs. It should be noted that the clonogenic survival rate depends on the cell population conditions during irradiation, but is independent of the time course of PC and CSC fractions after irradiation. Based on the experimental report evaluating the values of the coefficients to dose and dose square in the LQ model, it was assumed that the values of (α_0_, *β*_0_) for CSCs were lower than those for PCs in this study [2]. Under the constraints, the surviving fraction for the PCs and the CSCs can be expressed as:

(3) $$-\;\ln\;S_{\mathrm{PC}}\left(D\right)=\left(\alpha_{0\;\mathrm{PC}}\;+\;\frac{y_{D}}{\rho \pi r_{d}^{\;2}}\beta_{0\;\mathrm{PC}}\right)D\;+\;\beta_{0\;\mathrm{PC}}D^{2}$$

(4) $$-\;\ln\;S_{\mathrm{CSC}}\left(D\right)=\left(\alpha_{0\;\mathrm{CSC}}\;+\;\frac{y_{D}}{\rho \pi r_{d}^{\;2}}\beta_{0\;\mathrm{CSC}}\right)D\;+\;\beta_{0\;\mathrm{CSC}}D^{2},$$

where *S*_PC_ (*D*), *α*_0 PC_ and *β*_0 PC_ are the surviving fraction and the set of the model parameters (*α*_0_, *β*_0_) for the PCs, respectively; *S*_CSC_ (*D*), *α*_0 CSC_ and *β*_0 CSC_ are the surviving fraction and set of the model parameters (*α*_0_, *β*_0_) for the CSCs, respectively. Here, stochastically considering the surviving fraction for the cell population containing the PCs and the CSCs, the overall cell survival as a function of absorbed dose *S* (*D*) can be expressed by

(5) $$S\left(D\right)=\;S_{\mathrm{PC}}\;\left(D\right)\;\times \;f_{\mathrm{PC}}\;+\;S_{\mathrm{CSC}}\;\left(D\right)\;\times \;f_{\mathrm{CSC}}.$$

In this study, we used Eqs 3–5 for evaluating the impact of CSCs on the dose–response curve.

#### Determination of the model parameters and description of the cell survival curve

Two cell-killing models were used for describing the dose–response curve: one for the LQ model (Eq. 1), and the other one is the modified IMK model considering the CSC fraction (Eqs 3–5). On one hand, the set of the LQ parameters *θ* = (*α*, *β*) were determined by fitting the LQ model to the experimental cell survival curve using the least-square method and generating a random number for each parameter, and the survival curve was described based on Eq. 1. The summation of the square deviation of logarithmic survival values $\ell \text{(}d_{\text{e}}\text{|}\theta \text{)}$ is given as:

(6) $$\ell (d_{e}|\theta )=\overset{N}{\underset{i=1}{\sum }}\left[{\left({\mathrm{Exp}}_{i}-{\mathrm{Cal}}_{i}\right)}^{2}\right],$$

where *N* is the number of experimental data, Exp_*i*_ is the *i*-th experimental survival, Cal_*i*_ is that calculated by the present model, and *d*_e_ is the set of experimental data. On the other hand, the model considering the CSC fraction (Eqs 3–5) is complex, so the set of the parameters in the proposed model were obtained following the three steps.

- The input parameter *y*_*D*_ for the 150 kVp X-rays was calculated by using two the Monte Carlo (MC) codes: Electron Gamma Shower ver. 5 (EGS5) [26] for the photon procedure (cut-off energy = 1.0 keV) and WLTrack [27] for the electron procedure (cut-off energy = 1.0 eV). After sampling the electron energy spectra generated by the 150 kVp X-rays irradiation at 1 mm depth from surface of water, the energy deposited in the site with 1.0 μm diameter was sampled uniformly along the electron track.
- Using the calculated *y*_*D*_ (4.68 ± 0.05 keV/μm), Eqs 3–5 and experimental data *d*_e_, the set of the parameters *θ* = (*α*_0 PC_, *β*_0 PC_, *α*_0 CSC_, *β*_0 CSC_, *f*_CSC_) were simultaneously determined by the least-square method with the MC technique (Eq. 6).
- The cell survival curve as a function of absorbed dose *D* was described according to Eqs 3–5.

It should be noted that the accuracy of the MC simulation for calculating the *y*_*D*_ value was checked in the comparison with the measured data reported by Okamoto *et al.* [25]: the *y*_*D*_ value for 200 kVp X-rays calculated in this study (4.45 ± 0.03 keV/μm) agreed with their value [25] (4.51 ± 0.05 keV/μm).

#### Fit quality

We used the chi-square (χ^2^) value for evaluating the fit quality of the models used in this study. The χ^2^ value is defined as

(7) $$\chi^{2}=\frac{1}{N}\overset{N}{\underset{i=1}{\sum }}\frac{{\left(S_{i\;\exp}-S_{i\;\mathrm{model}}\right)}^{2}}{\sigma_{i\;\exp}^{2}},$$

where *N* is the number of the experimental data, *S*_exp_ is measured cell survival, *S*_model_ is cell survival estimated by the present model, *σ*_exp_ is the standard deviation of the measured cell survival.

#### Biologically effective dose estimation

The biologically effective dose (BED) is the index widely used for evaluating the curative effects in radiotherapy [28]. The BED defined in the LQ model is given by

(8) $$\mathrm{BED}=nd\left(1\;+\;\frac{d}{\alpha /\beta }\right),$$

where *d* is the absorbed dose per fraction and *n* is the number of fractions. To compare the BED calculated using the LQ model with that calculated using the proposed model, the *α*/*β* in the IMK model (Eq. 2) was calculated using the following equation:

(9) $$\frac{\alpha }{\beta }=\frac{\alpha_{0}\;}{\beta_{0}}+\;\frac{y_{D}}{\rho \pi r_{d}^{2}}.$$

The *α*/*β* for the overall cell populations with the PCs and CSCs cannot be deduced because the cell survival formula is complex (Eqs 3–5). For this reason, in this study the *α*/*β* values for the PCs and the CSCs were calculated separately, and we calculated the BED values for the PCs and CSCs.

## RESULTS

### Dose–response curve fitting by the conventional model and the proposed model

**Fig. 2.**
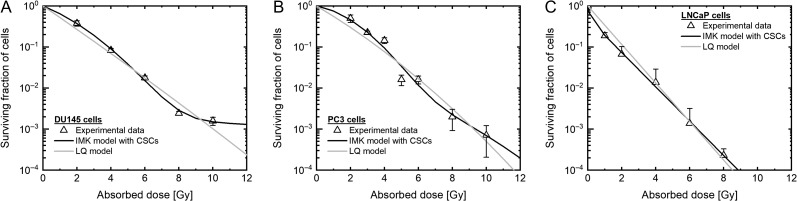
The dose–response curve in PCa cells on the log scale. The dose–response curve estimated by each model (A) in DU145 cells, (B) in PC3 cells, and (C) in LNCaP cells on the log scale. The triangle plot shows the measured value. The black line shows the data estimated by the IMK model with CSCs. The gray line shows the data estimated by the LQ model. PCa: prostate cancer, IMK: integrated microdosimetric kinetic, CSCs: cancer stem cells.

The survival rates of DU145 cells, PC3 cells and LNCaP cells were evaluated using the LQ model (Eq. 1) and the modified IMK model with the CSCs fraction (Eqs 3–5). The model parameters are listed in Table 1, and the cell survival curves described by the models are shown in Fig. 2. As shown in Fig. 2A and B, the measured cell survivals of the DU145 and the PC3 cells are subtly higher than those predicted by the LQ model at 10 Gy. In addition, the LNCaP cells exhibit higher radiosensitivity in the low-dose range <4 Gy, subtly lower radiosensitivity at 8 Gy, in comparison with the LQ model (Fig. 2C).

**Table 1.** Model parameters in the LQ model and the IMK model with the CSC fraction

		Cell line type		
Model type	Parameters	DU145	PC3	LNCaP
LQ model	*α* [Gy^-1^]	0.658 ± 0.005	0.551 ± 0.012	1.081 ± 0.002
	*β* [Gy^-2^]	0.002 ± 0.001	0.021 ± 0.001	
IMK model with CSCs	*α*_0PC_ [Gy^-1^]	0.332 ± 0.045	0.076 ± 0.013	8.931 ± 1.225
	*β*_0PC_ [Gy^-2^]	0.055 ± 0.007	0.108 ± 0.003	
	*α*_0CSC_ [Gy^-1^]	0.007 ± 0.006	0.065 ± 0.026	0.963 ± 0.001
	*β*_0CSC_ [Gy^-2^]	0.002 ± 0.003	0.025 ± 0.002	
	*f*_CSC_ × 100 [%]	0.183 ± 0.134	1.984 ± 0.055	50.34 ± 0.326
	$\frac{y_{D}}{\rho \pi r_{d}^{\;2\;}[\mathrm{Gy}]}$	0.954	0.954	0.954

The values in the parameters are mean ± standard errors.

**Fig. 3.**
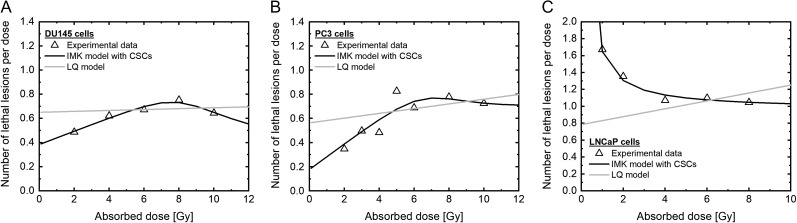
The dose–response curve in PCa cells on the linear scale. The dose–response curve estimated by each model (A) in DU145 cells, (B) in PC3 cells and (C) in LNCaP cells on the linear scale. The triangle plot shows the measured value. The black line shows the data estimated by the IMK model with CSCs. The gray line shows the data estimated by the LQ model. PCa: prostate cancer, IMK: integrated microdosimetric kinetic, CSCs: cancer stem cells.

In contrast, the model developed in this study generates the sigmoid-like curves (solid line in Fig. 2) with better agreement with the experimental data from the deduced χ^2^ values listed in Table 2. To further discuss the model performance, we also evaluated the number of lethal lesions (LLs) per dose ((−log*S*)/*D*), which enabled us to judge whether the model could reproduce the experimental data or not. Similarly, the proposed model was able to reproduce well the experimentally observed trends in the number of LLs per dose, as shown in Fig. 3.

**Table 2.** Chi-square value in comparison between the model and experiments

Cell line type	LQ model	IMK model with CSCs
DU145	9.723	0.826
PC3	19.54	2.697
LNCaP	1.362	0.0172

From the model analysis shown in Figs 2 and 3 and Table 2, the IMK model considering the CSCs shows the more precise dose–response curve for each cell line, compared with the prediction by the LQ model. Thus, it is suggested that the lower radiosensitivity at ~10 Gy is explainable by considering the effect of the small subpopulation of CSCs in dose–response curve.

### Prediction of the subpopulation of the CSCs with radioresistance

**Fig. 4.**
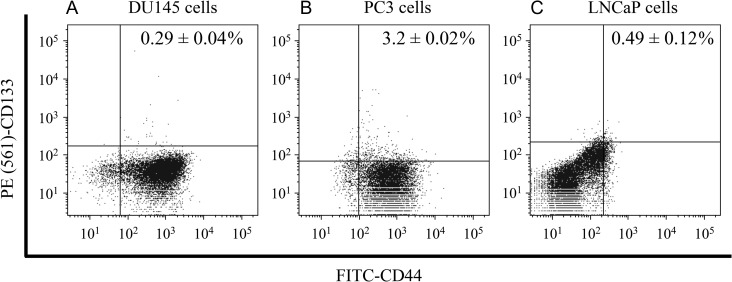
The CSC population in PCa cells. The fraction of cells expressing CD44 and CD133 markers was analyzed by flow cytometry. Representative histograms and dot plots of (A) DU145 cells, (B) PC3 cells and (C) LNCaP cells, illustrating the identification of multipositive cells. CD44- and CD133-positive cells were subtracted from their respective isotype controls. PCa: prostate cancer.

To validate the assumption that the cell population of the tumor is composed of PCs and CSCs, the markers for CSCs—FITC-CD44 and PE (561)-CD133—were measured by flow cytometry. The percentages of double-positive cells in DU145 cells, PC3 cells and LNCaP cells were 0.29 ± 0.05%, 3.20 ± 0.02% and 0.49 ± 0.12%, respectively (Fig. 4). Comparing these percentages with the CSC percentages estimated by the IMK model with CSCs (Table 1), the model-predicted percentages of the CSCs in DU145 cells and PC3 cells showed good agreement with the experimental values. In contrast, the percentage obtained by the model in LNCaP cells differed substantially from the experimental value, suggesting that the CSCs do not contribute to the modification of the dose–response curve in the case of LNCaP cells.

**Fig. 5.**
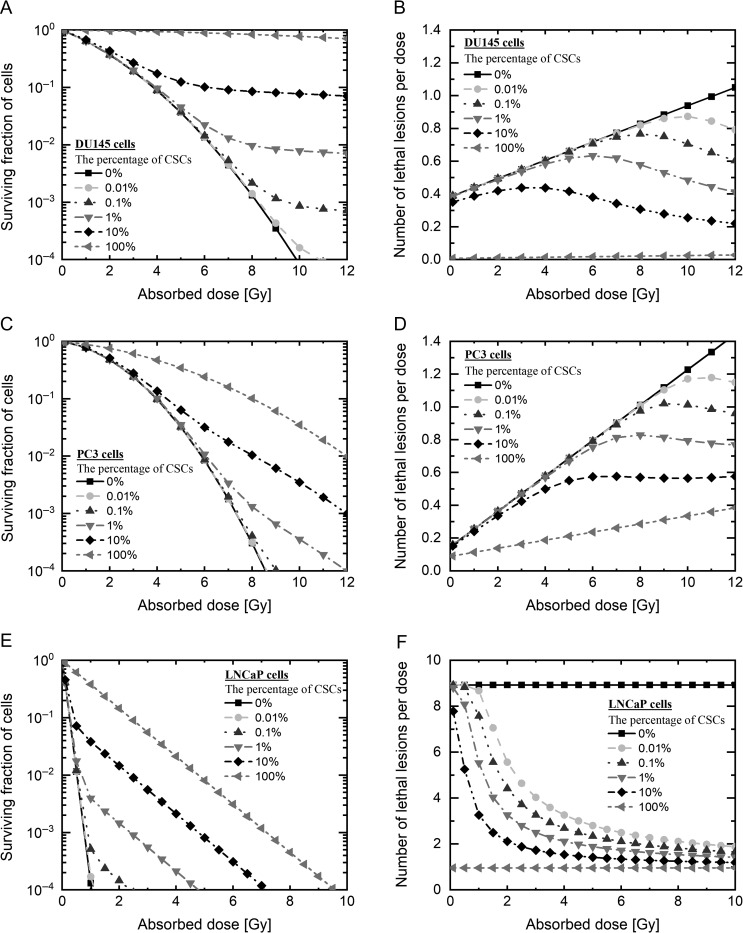
Prediction of the dose–response curve in PCa cells. Prediction of the dose–response curve by each model (A) in DU145 cells, (C) in PC3 cells and (E) LNCaP cells, shown on the log scale, and (B) DU145 cells, (D) PC3 cells and (E) LNCaP cells, shown on the linear scale. The black line with squares shows the survival data on the assumption that 0% of radioresistant cells were present, estimated by the IMK model with CSC prediction; the gray broken line with circles shows 0.01% radioresistant cells, the dark gray broken line with triangles shows 0.1% radioresistant cells, the gray broken line with inverted triangles shows 1% radioresistant cells, the black broken line with diamonds shows 10% radioresistant cells, and the gray broken line with left-pointing triangles shows 100% radioresistant cells. PCa: prostate cancer, IMK: integrated microdosimetric kinetic, CSCs: cancer stem cells.

Based on the model validation shown in Fig. 2, we additionally estimated the dose–response curve as a function of the CSC fraction *f*_CSC_ by using Eqs (3–5) for each cell line. As increased with the CSC fraction, the degree of radioresistance becomes especially high in the high dose range (Fig. 5A, C and E). The numbers of nuclear LLs per dose were predicted for different contents of CSCs, and are shown in Fig. 3. As shown in the right-hand panel of Fig. 5, it appears that the even a small percentage of the CSCs can modify the dose–response curve. This applies to the three types of PCa cells used in this study.

**Fig. 6.**
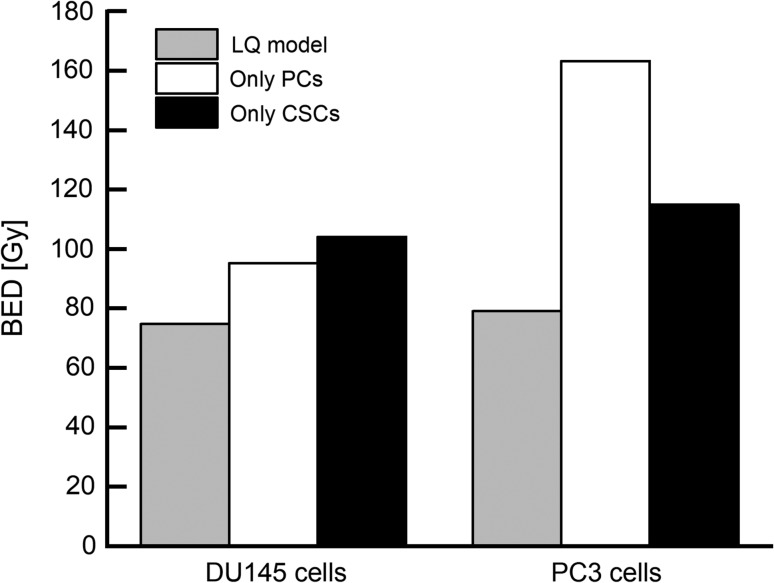
Prediction of the BED by each model. The estimation of BED in DU145 cells and PC3 cells by each model. The assumed irradiation method was 37 fractions × 2 Gy. This is a general radiotherapy method for PCa in Japan. BED: biologically effective dose, PCa: prostate cancer.

Finally, the BED used in radiotherapy was calculated for evaluating the impact of CSCs on the treatment planning. We assumed the fractionated regimen, i.e. 2 Gy × 37 fractions, and calculated the BED values by using the LQ model and the present model. Compared with the prediction by the LQ model, the biological dose was higher based on the IMK model, with CSCs, as shown in Fig. 6 (e.g. 95.33 Gy for the PCs by the IMK model, 75.85 Gy by the LQ model in the case of the DU145 cells). In the same manner as DU145 cells, the BED obtained by the IMK model for the CSCs of PC3 cells was higher than that obtained by the LQ model (161.3 Gy for PCs by the IMK model, 79.15 Gy by the LQ model). The higher BED values for both PCs and CSCs are predominantly attributed to the reduced *α* and increased *β* obtained from the fitting approach. Comparing the value for PCs with that for CSCs, the BED of CSCs was lower than that of the PCs (115.1 Gy for CSCs by the IMK model). We cannot interpret this tendency because the underlying mechanisms remain unclear. Further investigations by *in vitro* studies and use of models are necessary for clarifying the mechanisms such as DNA repair function and so on.

## DISCUSSION

The stem-like cell population was incorporated into the IMK model in this study, resulting in good agreement with *in vitro* survival rate in DU145 cells and PC3 cells, as shown in Fig. 2 and Table 2. The CSC fraction estimated by the modified IMK model (Table 1) and the experimental data by the flow-cytometric analysis (Fig. 4) corresponded well in the case of the DU145 and PC3 cell lines. Focusing on the predicted CSC contents of PC3 and LNCaP cells, it can be pointed out that there is room for improving the proposed model, as well as for improving how to determine the model parameters associated with the CSCs.

**Fig. 7.**
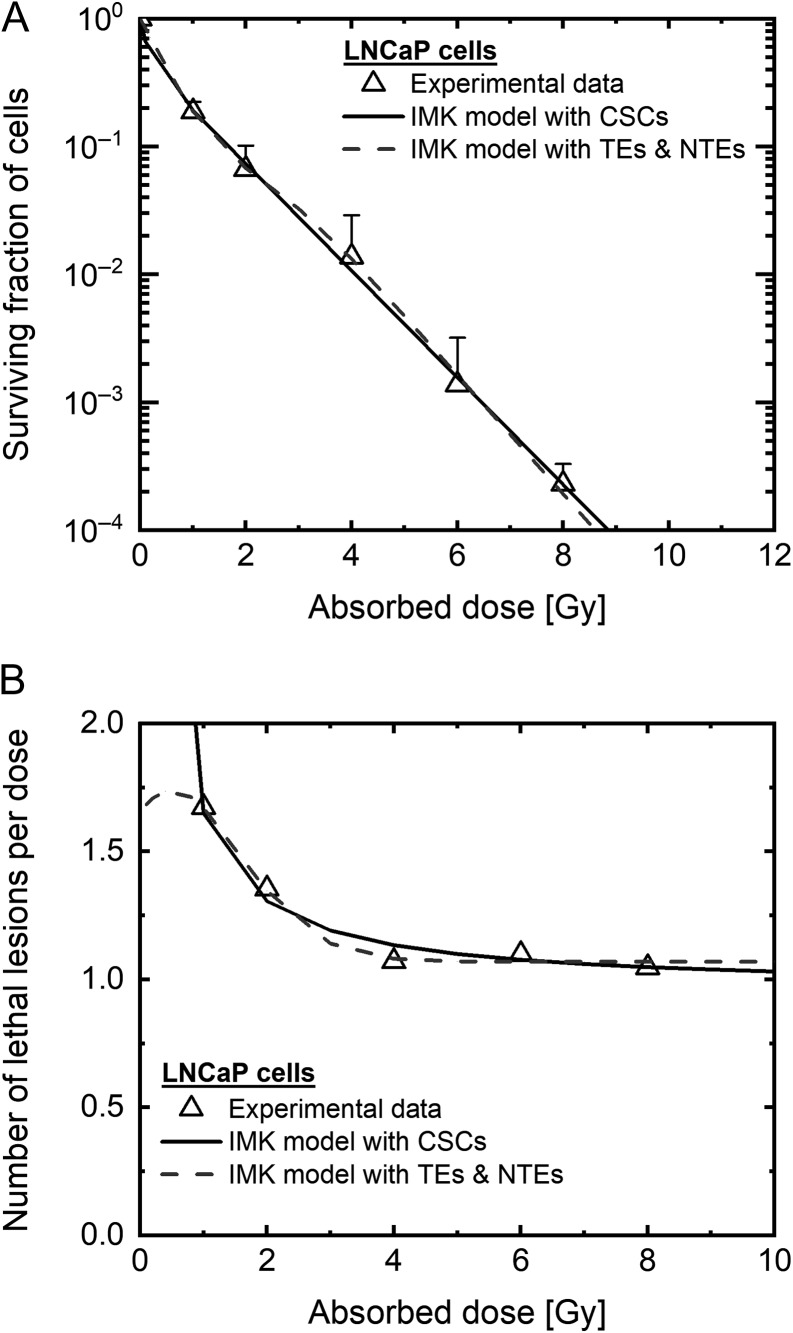
Prediction of the dose–response curve in LNCaP cells. Dose–response curve estimation by the IMK model with TEs and NTEs in LNCaP cells on (A) the log scale and (B) the linear scale. The black broken line shows the survival data estimated by the IMK model with TEs and NTEs. Other data are as shown in Fig. 2E and F. IMK: integrated microdosimetric, TEs: targeted effects, NTEs: non-targeted effects.

For the LNCaP cells, there was a large discrepancy between the model results and the experimental results. Focusing on the comparison of the CSC fractions, the dominant factor to induce the sigmoid curve might not be the existence of a subpopulation of CSCs. So here we added the IMK model analysis in consideration of both the DNA-TEs and the non-targeted effects (NTEs), because of higher radiosensitivity in the low dose range <4 Gy. The cell survival formula for the NTEs according to the IMK model [22] is given by:

(10) $$-\;\ln\;S_{\mathrm{NT}}=\delta [1-e^{-(\alpha_{b}+\gamma \beta_{b})D-\beta_{b}D^{2}}]e^{-(\alpha_{b}+\gamma \beta_{b})D-\beta_{b}D^{2}},$$

where *S*_NT_ is the surviving fraction for NTEs, *δ* is the maximum number of LLs per cell nucleus induced in non-irradiated cells, and *α*_b_ and *β*_b_ are the proportionality factors for the NTEs to *D* [Gy] and *D*^2^ [Gy^2^], respectively, which represent the target activation probabilities for the cells hit by the radiation releasing cell-killing signals [22]. Thus, the cell surviving fraction considering the DNA-TEs and the NTEs is given by:

(11) $$S=S_{T}\;\times S_{\mathrm{NT}},$$

where *S*_T_ is the surviving fraction for the DNA-TEs, which is given by Eq. 2. Figure 7 shows the fitting results of the IMK model for DNA-TEs and NTEs [22], in which the NTEs might contribute to modifying the dose–response curve of the LNCaP cells.

The previous study showed that prostatospheres with a high level of CD133 expression have a highly efficient DNA repair function and resistance to damage induction by reactive oxygen species (ROS) [29]. The ROS is the main indirect effector that is exerting a cell-killing effect as a result of the ionizing radiation [30, 31]. Since high capacity to diminish the ROS in the CSCs is intricately related to radioresistance [32], the presence of the CSCs might play an important role in the change in radiosensitivity.

In this study, the model prediction (Fig. 5) shows that radioresistance increases with increase in the percentage of CSCs, and that a cell population composed of 100% CSCs exhibits the most radioresistance. A few reports have shown that cancer cells can acquire apoptosis resistance as the percentage of CSCs increases [33]. However, resistance to apoptosis increases in CSCs when in the co-culture of CSCs and PCs configuration [33, 34]. This phenomenon is postulated to be induced by virtue of intercellular communication, such as bystander effects between CSCs and non-CSCs. Although the cell-killing signal is a well-known pattern in bystander effects, the recent microbeam studies have revealed that the cell-killing signal weakens when only the CSCs are irradiated [35]. Thus suggests that it is necessary to quantitatively evaluate whether the change in radiosensitivity depends on the proportion of the CSCs or not. In addition, the mechanisms for inducing the increase in the CSC markers during the fractionated irradiations remain unclear in this study. A clinically relevant daily fractionated regimen (i.e. 2.0 Gy per fraction at 24-h intervals) can induce the increased expression of CSC markers and epithelial–mesenchymal transition in prostate cancer cells [36], thereby making cancer cells a more heterogeneous cell population with lower radiosensitivity. These findings might be incorporated into the BED calculation in the future. Indeed, the BED value estimated by the IMK model with CSCs was higher than that estimated by the LQ model (Fig. 5). In view of this finding, it was suggested that administration of higher absorbed doses would be necessary to control the radioresistant cells in order to avoid recurrence or metastasis. Therefore, the conventional planning method in radiotherapy includes the possibility that a sufficient dose might not be administered. To clarify this concern, to determine what dose is sufficient, some additional clinical research is necessary.

## CONCLUSION

In conclusion, the *in vitro* experiments and the IMK model study provide a more precise dose–response curve as a function of absorbed dose and the contents of CSCs. The CSC marker might be useful as one indicator to quantify the degree of modification of the cell survival curve and the BED for PCs and CSCs, and treatment planning in radiotherapy can be improved by using the developed cell-killing model, which takes the CSC fraction into account. The findings in this study contribute to an increased understanding of the relationship between the fraction of CSCs and radioresistance in radiation therapy for prostate cancer.
